# The Relationship between Waterpipe and Cigarette Smoking in Low and Middle Income Countries: Cross-Sectional Analysis of the Global Adult Tobacco Survey

**DOI:** 10.1371/journal.pone.0093097

**Published:** 2014-03-24

**Authors:** Mohammed Jawad, John Tayu Lee, Christopher Millett

**Affiliations:** 1 Department of Primary Care and Public Health, School of Public Health, Imperial College London, London, United Kingdom; 2 South Asia Network for Chronic Disease, Public Health Foundation of India, New Delhi, India; Universität Bochum, Germany

## Abstract

**Introduction:**

Waterpipe tobacco smoking is receiving growing attention due to accumulating evidence suggesting increasing prevalence in some populations and deleterious health effects. Nevertheless, the relationship between waterpipe and cigarette smoking remain unknown, particularly in low and middle income countries.

**Materials and Methods:**

We analysed waterpipe and cigarette smoking using data from Global Adult Tobacco Survey, a household survey of adults aged ≥15 years conducted between 2008–2010 in LMICs. Factors associated with waterpipe and cigarette use were assessed using multiple logistic regression. Factors associated with the quantity of waterpipe and cigarette smoking were assessed using log-linear regression models.

**Results:**

After adjusting for age, gender, residence, education, occupation and smokeless tobacco use, waterpipe smoking was significantly higher among cigarette users than in non-cigarette users in India (5.6% vs. 0.6%, AOR 13.12, 95% CI 7.41–23.23) and Russia (6.7% vs. 0.2%, AOR 27.73, 95% CI 11.41–67.43), but inversely associated in Egypt (2.6% vs. 3.4%, AOR 0.21, 95% CI 0.15–0.30) and not associated in Vietnam (13.3% vs. 4.7%, AOR 0.96, 95% CI 0.74–1.23). Compared to non-cigarette smokers, waterpipe smokers who also used cigarettes had more waterpipe smoking sessions per week in Russia (1.3 vs. 2.9, beta coefficient 0.31, 95% CI 0.06, 0.57), but less in Egypt (18.2 vs. 10.7, beta coefficient −0.45, 95% CI −0.73, −0.17) and Vietnam (102.0 vs. 79.3, beta coefficient −0.31, 95% CI −0.56, −0.06) and similar amounts in India (29.4 vs. 32.6, beta coefficient −0.12, 95% CI −0.46, 0.22).

**Conclusions:**

Waterpipe smoking is low in most LMICs but important country-level differences in use, including concurrent cigarette smoking, should be taken into account when designing and evaluating tobacco control interventions.

## Introduction

Waterpipe tobacco smoking (hookah, shisha, narghile) has recently received increased attention from public health researchers and practitioners due to its growing use in some settings [1]. Studies suggest that young people and adolescents may be increasingly attracted to this smoking method, which may act as a precursor of future cigarette use [2], [3]. In US high school students, waterpipe smoking prevalence increased by 18% between 2008 and 2010 [4], and in Syrian high school students, 42% over the same time period [2]. In one United Kingdom university, over half of medical students had tried waterpipe smoking [5]. Despite the growing attention on waterpipe tobacco smoking worldwide [6] little is known about the relationship between waterpipe and cigarette smoking, particularly in low and middle income countries where waterpipe smoking may be more common.

This growing use may be due in part to a widespread public perception that waterpipe smoking is less harmful, and thus more socially acceptable, than cigarettes [7], despite exhibiting similar adverse health effects. Lung cancer, respiratory illness, low birth weight and periodontal disease have been significantly associated with waterpipe smoking [8]. Analyses of urinary cotinine levels among daily users suggest that one waterpipe session may equate to ten cigarettes' worth of nicotine [9] which may lie above the “addiction threshold” [10] and subject users to dependency and failed quit attempts. Sharing waterpipe between peers is a common practice and has been implicated in the transfer of infectious diseases [11], [12].

Improving understanding of the epidemiology of waterpipe tobacco smoking was a key recommendation of the World Health Organisation (WHO) in 2005 [13]. Questions on waterpipe use are not generally included in routine surveillance on tobacco resulting in very little population level data being available in most countries. While waterpipe prevalence has been documented in several regions, little is known on the patterns of waterpipe in relation to other tobacco products, including how volume use patterns vary with demographic variables [14]–[16]. As the tobacco industry is aggressively targeting low and middle income countries (LMICs) as growth markets, it is important to understand how their efforts are influencing different types of tobacco use, including waterpipe smoking. We therefore examined the prevalence and factors associated with waterpipe smoking, including the relationship between waterpipe and cigarette use among adults in all LMICs who participating in the Global Adult Tobacco Survey (GATs).

## Materials and Methods

### Sample and data

This study used data from the Global Adult Tobacco Survey (GATS) which was conducted in multiple countries during 2008–2010. GATS is considered to be the global standard for monitoring adult tobacco use and a key tobacco control indicator, and a standard protocol and questionnaire were used in participating countries [17]. It employs multi-stage geographically clustered sample design to produce nationally representative estimates for adults population aged 15 years and over [18]. For the purpose of the study, we used data from the 13 LMICs in GATS that included questions of waterpipe smoking, which were freely available on the Centre for Disease Control and Prevention website (Global Tobacco Surveillance System Data: http://nccd.cdc.gov/GTSSData/Ancillary/DataReports.aspx?CAID=2). These included Bangladesh, Brazil, China, Egypt, India, Mexico, Philippines, Russia, Thailand, Turkey, Ukraine, Uruguay and Vietnam.

### Outcome measures

The two main outcome measures were whether the respondent was a current waterpipe/cigarette user and the weekly volume of waterpipe/cigarette use. In the GATS, participants were asked the following question: “on average, how many waterpipe sessions/cigarettes do you currently smoke per day/per week/during a usual week?” We defined respondents as current waterpipe/cigarette smokers if they smoked at least once per week. We defined respondents as dual smokers if they are categorised as both waterpipe and cigarettes smokers. For the purpose of this study, countries with a current waterpipe smoking prevalence of ≥1.0% or ≥100 current users were included for further analysis. Only four of the 13 countries met these criteria: Egypt, India, Russia and Vietnam. Removing observations with missing data in outcome or control variables (0.3% of sample size) resulted in a final sample size of 111,253 respondents (sample size in Egypt 20,914, India 69,030, Russia 11,388 and Vietnam 9,921).

### Statistical analysis

We assessed the association between demographic and socioeconomic factors with waterpipe smoking and dual waterpipe and cigarette smoking using multiple logistic regression. Our model included age (grouped into <30, 30–50, >50 years), gender, residence (urban, rural), education (less than primary, completed primary, completed secondary, completed higher than secondary), occupation (employed, unemployed, ‘other’ e.g. retired, student, homemaker), daily or non-daily smokeless tobacco use (yes, no) and current cigarette use (yes, no). As there were very few respondents in several education and occupation category options in Russia, we categorised the education variable into two groups (completed secondary or less; completed higher than secondary) and omitted occupation in our analyses. To look at the association between waterpipe and cigarette use, we included current waterpipe use as a predictor variable for cigarette use, and vice versa. We ran separate analyses for each country and reported adjusted odds ratios (AORs) with a 95% confidence interval.

To reduce skewness for our continuous outcome variables, the volume of waterpipe/cigarette smoking for users was transformed using the logarithm function. The association between variables mentioned above and level of consumption of tobacco product use was estimated using a log-linear regression model. Therefore, the results from our model can be interpreted as the percentage change of amount of waterpipe/cigarette smoking in relation to one unit change in predictor variables.

We tested for multicollinearity for covariates controlled for in our analysis. The multicollinearity diagnostics (variance inflation factor, VIF) were all less than 5, indicating that the assumption of reasonable independence among predictor variables was met. Sampling weights were used to account for the complex, multi-stage design of the GATS survey. We performed the statistical analyses using Stata 13.1 (StataCorp).

## Results

### Prevalence and weekly volume use of current waterpipe smoking

Compared to cigarettes, the prevalence of waterpipe smoking was low in GATS countries. A prevalence of <0.5% was reported in Mexico (0.0%), Philippines (0.0%), Thailand (0.0%), Brazil (0.1%), Uruguay (0.1%) and China (0.4%). Slightly higher was the prevalence reported by Bangladesh (0.7%), Turkey (0.7%), India (0.8%) and Ukraine (0.9%). Those with a prevalence of ≥1.0% included Russia (2.7%, 95% CI = 2.0–3.3%), Egypt (3.3%, 95% CI = 2.9–3.6%) and Vietnam (6.4%, 95% CI = 5.5–7.3%). The prevalence of dual waterpipe and cigarette smoking was 2.6% (95% CI = 1.9–3.1%) in Russia, 0.4% (95% CI = 0.3–0.5%) in Egypt, and 2.6% (95% CI = 2.1–3.0%) in Vietnam and 0.3% (95% CI = 0.2–0.4%) in India.

Population estimates for prevalence and volume of waterpipe and cigarette smoking by socio-demographic characteristics for the four countries that had a prevalence of ≥1% or ≧ 100 current users (India, Russia, Egypt and Vietnam) are presented in Table 1.

**Table 1:** Current prevalence of waterpipe and cigarette smoking, and frequency of use among users in India, Russia, Egypt and Vietnam.

	India (N = 69030)				Russia (N = 11388)				Egypt (N = 20914)				Vietnam (N = 9921)			
	Waterpipe		Cigarettes		Waterpipe		Cigarettes		Waterpipe		Cigarettes		Waterpipe		Cigarettes	
	%	No/W	%	No/W	%	No/W	%	No/W	%	No/W	%	No/W	%	No/W	%	No/W
Overall (≥15 years)	0.8	30.5	4.8	26.4	2.7	2.8	38.0	105.4	3.3	17.2	16.3	130.3	6.4	92.9	19.2	76.5
**Age**																
<30	0.3	27.4	3.6	18.1	6.2	2.5	44.7	90.5	1.3	15.1	12.4	128.6	3.6	67.7	14.1	62.3
30–50	0.9	33.6	6.4	29.4	2.3	3.0	45.9	108.7	4.7	17.5	20.3	132.1	8.5	96.7	24.7	80.3
>50	2.0	28.6	4.7	31.0	0.3	6.7	24.7	120.0	5.4	18.2	17.9	129.0	7.0	105.0	17.3	85.1
**Gender**																
Male	1.0	30.7	8.9	26.3	3.5	3.4	59.0	119.6	6.1	16.8	31.7	130.6	13.0	93.2	38.5	76.9
Female	0.6	30.2	0.5	28.6	2.0	2.0	20.7	71.8	0.3	27.3	0.2	74.7	0.0	65.1	1.0	60.6
**Residence**																
Urban	0.0	27.3	6.5	29.5	3.2	2.4	39.0	102.6	2.4	15.3	17.3	132.4	2.5	86.5	21.6	79.0
Rural	1.1	30.8	4.2	24.4	1.1	6.7	35.1	114.7	4.0	18.2	15.4	128.3	8.1	93.8	18.2	75.2
**Education**																
Less than primary	1.4	31.0	3.6	25.3	1.3	3.4	34.8	107.8	5.1	18.4	17.1	129.8	5.0	106.9	18.7	97.5
Primary	0.6	29.2	5.7	25.2	3.4	2.7	39.5	104.5	3.2	15.0	18.1	130.3	7.7	92.5	19.6	71.3
Secondary	0.0	31.1	5.9	29.1	-	-	-	-	0.0	10.0	11.8	126.3	5.4	80.1	19.1	72.9
Higher than secondary	0.0	27.1	5.9	28.3	-	-	-	-	2.0	15.7	15.8	131.2	2.5	81.3	18.3	63.2
**Occupation**																
Employed	1.0	28.1	8.9	27.1	2.7	2.8	38.0	105.4	6.3	16.7	31.6	132.0	7.9	92.3	24.3	77.1
Unemployed	0.0	31.3	6.2	25.0	0.0	-	0.0	-	0.9	18.6	3.5	121.9	3.9	82.8	10.6	70.9
Other	0.8	31.5	3.4	25.9	0.0	-	0.0	-	1.1	21.5	5.8	119.0	2.0	105.8	4.4	68.2
**Smokeless tobacco**																
Current non-user	0.7	28.2	3.8	30.4	2.6	2.9	37.9	105.5	3.2	17.1	14.9	132.1	6.5	92.6	19.4	76.7
Current user	1.0	35.5	7.8	20.7	20.7	2.2	63.8	99.2	8.1	19.4	74.3	114.0	0.0	206.3	7.6	44.6
**Waterpipe smoking**																
Current non-user	-	-	4.6	26.0	-	-	36.4	106.1	-	-	16.4	130.1	-	-	17.8	80.7
Current user	-	-	33.3	33.8	-	-	95.1	96.0	-	-	13.1	135.4	-	-	40.0	49.0
**Cigarette smoking**																
Current non-user	0.6	29.4	-	-	0.2	1.3	-	-	3.4	18.2	-	-	4.7	102.0	-	-
Current user	5.6	32.6	-	-	6.7	2.9	-	-	2.6	10.7	-	-	13.3	79.3	-	-

Note: % = prevalence of current (daily or weekly) smoking, No/W = mean number waterpipe sessions/cigarettes per week among current users; Russia education: 1) Completed secondary or less; 2) Completed higher than secondary; Russia occupation: omitted due to very low/no respondents in other categories.

### Prevalence and factors associated with current waterpipe smoking

Table 2 presents factors associated with waterpipe/cigarette/dual waterpipe and cigarette smoking. India: The prevalence of waterpipe smoking was significantly higher in those aged over 50 years (2.0% vs. 0.3% compared with those aged under 30 years), in those living in rural compared with urban areas (1.1% vs. 0.0%), in those with lower educational attainment (1.4% vs. 0.0%) and among current cigarette smokers compared with non-cigarette smokers (5.6% vs. 0.6%; AOR = 13.12, 95% CI = 7.41–23.23). Cigarette prevalence was significantly higher in those aged over 50 years (4.7% vs. 3.6% compared with those aged under 30 years), among males (8.9% vs. 0.5%) and in those living in urban areas (6.5% vs. 4.2%). Those with completion of secondary education were more likely to be cigarette smokers than those with less than primary education (5.9% vs. 3.6%), as were those in current employment (8.9% vs. 6.2%), users of smokeless tobacco as opposed to non-users (7.8% vs. 3.8%; AOR = 1.66, 95% CI = 1.44–1.93), and current waterpipe smokers as opposed to non-waterpipe smokers (33.3% vs. 4.6%; AOR = 14.34, 95% CI = 7.68–26.77). The likelihood of dual waterpipe and cigarette smoking was higher in those aged more than 50 years (AOR = 2.26, 95% CI = 1.23–4.14, compared with those aged less than 30), and in users of smokeless tobacco (AOR = 4.56, 95% CI = 2.20–9.43).

**Table 2:** Factors associated with waterpipe tobacco and cigarette smoking among current users in India, Russia, Egypt and Vietnam.

	India			Russia			Egypt			Vietnam		
	Waterpipe	Cigarettes	Dual Waterpipe and Cigarettes	Waterpipe	Cigarettes	Dual Waterpipe and Cigarettes	Waterpipe	Cigarettes	Dual Waterpipe and Cigarettes	Waterpipe	Cigarettes	Dual Waterpipe and Cigarettes
**Age**												
<30	1	1	1	1	1	1	1	1	1	1	1	1
30–50	2.29 (1.35, 3.90)	1.80 (1.53, 2.11)	1.39 (0.72, 2.71)	0.34 (0.23, 0.48)	1.23 (1.05, 1.43)	0.68 (0.15, 3.08)	2.75 (1.95, 3.90)	1.45 (1.25, 1.69)	0.87 (0.46, 1.65)	2.34 (1.64, 3.33)	1.54 (1.25, 1.91)	1.37 (0.88, 2.14)
>50	5.30 (3.21, 8.74)	1.38 (1.14, 1.68)	2.26 (1.23, 4.14)	0.08 (0.03, 0.24)	0.51 (0.43, 0.59)	-	2.93 (1.94, 4.40)	1.35 (1.12, 1.62)	0.60 (0.23, 1.54)	2.64 (1.79, 3.89)	1.45 (1.13, 1.85)	1.19 (0.72, 1.97)
**Gender**												
Male	1	1	1	1	1	1	1	1	1	1	1	1
Female	0.81 (0.44, 1.47)	0.06 (0.04, 0.08)	1.56 (0.73, 3.33)	1.23 (0.86, 1.78)	0.18 (0.15, 0.21)	0.75 (0.35, 1.61)	0.04 (0.02, 0.09)	0.01 (0.00, 0.01)	0.02 (0.00, 0.13)	0.01 (0.01, 0.02)	0.01 (0.01, 0.02)	-
**Residence**												
Urban	1	1	1	1	1	1	1	1	1	1	1	1
Rural	4.21 (2.53, 7.00)	0.60 (0.52, 0.70)	1.38 (0.72, 2.63)	0.35 (0.22, 0.55)	0.87 (0.76, 0.99)	3.11 (0.57, 16.99)	1.40 (1.11, 1.75)	0.74 (0.66, 0.83)	1.39 (0.83, 2.32)	3.35 (2.44, 4.61)	0.56 (0.47, 0.66)	2.26 (1.56, 3.28)
**Education**												
Less than primary	1	1	1	1	1	1	1	1	1	1	1	1
Primary	0.52 (0.35, 0.77)	1.29 (1.10, 1.52)	0.98 (0.59, 1.63)	-	-	-	0.61 (0.42, 0.88)	0.82 (0.67, 1.01)	0.24 (0.08, 0.72)	1.51 (1.10, 2.09)	0.68 (0.55, 0.85)	2.59 (1.56, 4.29)
Secondary	0.26 (0.12, 0.56)	1.29 (1.06, 1.56)	0.45 (0.19, 1.10)	-	-	-	0.41 (0.24, 0.71)	0.71 (0.55, 0.92)	0.79 (0.21, 2.96)	1.25 (0.80, 1.95)	0.64 (0.48, 0.86)	2.75 (1.45, 5.25)
Higher than secondary	0.13 (0.06, 0.26)	1.16 (0.96, 1.40)	0.33 (0.15, 0.73)	2.03 (1.18, 3.50)	1.07 (0.94, 1.22)	1.27 (0.11, 14.79)	0.33 (0.26, 0.43)	0.60 (0.52, 0.69)	0.50 (0.26, 0.96)	0.60 (0.36, 1.00)	0.42 (0.31, 0.56)	1.06 (0.51, 2.20)
**Occupation**												
Employed	1	1	1	-	-	-	1	1	1	1	1	1
Unemployed	1.05 (0.55, 2.01)	0.65 (0.49, 0.86)	0.10 (0.04, 0.24)	-	-	-	0.58 (0.31, 1.09)	0.68 (0.53, 0.87)	0.73 (0.18, 2.95)	0.56 (0.33, 0.96)	0.32 (0.22, 0.46)	0.42 (0.14, 1.24)
Other	0.95 (0.62, 1.46)	0.63 (0.55, 0.73)	0.31 (0.17, 0.57)	-	-	-	0.34 (0.23, 0.50)	0.28 (0.22, 0.34)	0.14 (0.04, 0.56)	0.46 (0.30, 0.71)	0.17 (0.13, 0.23)	0.28 (0.13, 0.59)
**Smokeless tobacco**												
Current non-user	1	1	1	1	1	1	1	1	1	1	1	1
Current user	0.77 (0.51, 1.17)	1.66 (1.44, 1.93)	4.56 (2.20, 9.43)	5.37 (2.41, 11.94)	1.30 (0.43, 3.91)	-	1.93 (1.14, 3.28)	9.33 (6.28, 13.84)	1.32 (0.53, 3.32)	0.62 (0.16, 2.45)	1.92 (0.46, 8.06)	1.52 (0.28, 8.21)
**Waterpipe smoking**												
Current non-user	-	1	-	-	1	-	-	1	-	-	1	-
Current user	-	14.34 (7.68, 26.77)	-	-	32.93 (13.22, 82.02)	-	-	0.21 (0.14, 0.30)	-	-	0.96 (0.74, 1.23)	-
**Cigarette smoking**												
Current non-user	1	-	-	1	-	-	1	-	-	1	-	-
Current user	13.12(7.41, 23.23)	-	-	27.73(11.41, 67.43)	-	-	0.21 (0.15, 0.30)	-	-	0.96 (0.74, 1.23)	-	-

Note: AOR: adjusted odds ratios, CI: confidence interval; Current waterpipe/cigarettes: daily or weekly use.

Russia education: 1) Completed secondary or less; 2) Completed higher than secondary; Russia occupation: omitted due to very low/no respondents in other categories.

Female population were dropped when analysing dual waterpipe and cigarette smoking in Vietnam, as none of the female respondents were dual users of both products.

Russia: Waterpipe smoking was more prevalent in those aged under 30 years compare to over 50 years (6.2% vs. 0.3%), those in urban residence (3.2% vs. 1.1%), those with more educational attainment (3.4% vs. 1.3%;), those who use smokeless tobacco (20.7% vs. 2.6%; AOR = 5.37, 95% CI = 2.41–11.94) and those who smoke cigarettes (6.7% vs. 0.2%; AOR = 27.73, 95% CI = 11.41, 67.43). Compared to those aged under 30 years, cigarette smoking was significantly higher in those aged 30–50 years (45.9% vs. 44.7%), but lower in those aged over 50 years (24.7% vs. 44.7%). Cigarette smoking was also more prevalent in males (59.0% vs. 20.7%), those in urban residence (39.0% vs. 35.1%), and among waterpipe smokers (95.1% v 36.4%; AOR = 32.93, 95% CI = 13.22–82.02). There was no significant association between respondents' socio-demographic characteristics (including age, gender, and education) and the likelihood of dual waterpipe and cigarette smoking.

Egypt: Waterpipe smoking was significantly higher in those aged over 50 years (5.4% vs. 1.3% compared with those aged under 30 years), amongst males (6.1% vs. 0.3%), those in rural residence (4.0% vs. 2.4%), those with lower educational attainment (5.1% vs. 2.0% compared with those with the highest educational attainment), the employed (6.1% vs. 0.9% compared with those unemployed), smokeless tobacco users (8.1% vs. 3.2%; AOR = 1.93, 95% CI = 1.14–3.28), but lower for cigarette smokers (2.6% vs. 3.4%; AOR = 0.21, 95% CI = 0.15–0.30). Cigarette smoking was significantly higher in those aged over 50 years (17.9% vs. 12.4%, compared with those aged less than 30 years), males (31.7% vs. 0.2%), those in urban residence (17.3% vs. 15.4%), those with less educational attainment (17.1% vs. 15.8%, compared with those with the highest educational attainment), those employed (31.6% vs. 3.5%, compared with those unemployed), smokeless tobacco users (74.3% vs. 14.9%; AOR = 9.33, 95% CI = 6.28–13.84), but lower for waterpipe smokers (13.1% vs. 16.4%; AOR = 0.21, 95% CI = 0.14–0.30). The likelihood of dual waterpipe and cigarette smoking was lower in females (AOR = 0.02, 95% CI = 0.00–0.13) and those with higher education attainment (AOR = 0.50, 95% CI = 0.26–0.96 for those with higher education and above compared with those with no formal education).

Vietnam: Waterpipe smoking was more prevalent in those aged over 50 years (7.0% vs. 3.6%) amongst males (13.0% vs. 0.0%), those in rural residence (8.1% vs. 2.5%) and those employed (7.9% vs. 2.0%, compared with those unemployed). Similarly, cigarette smoking was significantly higher in those aged over 50 years (17.3% vs. 14.1%, compared with those aged less than 30 years), amongst males (38.5% vs. 1.0%) and the employed (24.3% vs. 10.6%, compared with those unemployed). However, it was higher among those in urban residence (21.6% vs. 18.2%) and those with less educational attainment (18.7% vs. 18.3%). The likelihood of dual waterpipe and cigarette smoking was higher among those in rural residence (AOR = 2.26, 95% CI = 1.56–3.28, compared with those in urban residence) and those with education attainment of primary and secondary school completed (AOR = 2.59, 95% CI = 1.56–4.29 for those with primary school completed compared with those with no formal education).

### Factors associated with consumption levels among current waterpipe smokers

The mean number of waterpipe sessions per week among current users was 2.8 in Russia, 17.2 in Egypt, 30.5 in India and 92.9 in Vietnam. Table 3 presents associations between volume of waterpipe/cigarette use and our predictor variables. The number of waterpipe sessions per week among current users in India was higher among those living in rural areas (coefficient = 0.60 [95% CI = 0.02, 1.17]) but this did not vary with other characteristics. In Russia, the number of waterpipe sessions per week was lower for females (coefficient = −0.30 [95%CI = −0.57, −0.04]), higher for those in rural residence (coefficient = 0.57 [95%CI = 0.16, 0.98]) and for those who smoked cigarettes (coefficient = 0.31 [95%CI = 0.06–0.57]). In Egypt, the number of waterpipe sessions per week was higher for users of smokeless tobacco (coefficient = 0.28 [95%CI = 0.01, 0.55]), but lower for current cigarette smokers (coefficient = −0.45 [95%CI = −0.73, −0.17]). In Vietnam, the number of waterpipe sessions per week was significantly higher in the older age groups and males but lower for cigarette smokers (coefficient = −0.31 [95%CI = −0.56, −0.06]).

**Table 3:** Factors associated with consumption levels among current waterpipe and cigarette smokers.

	India		Russia		Egypt		Vietnam	
	Waterpipe	Cigarettes	Waterpipe	Cigarettes	Waterpipe	Cigarettes	Waterpipe	Cigarettes
**Age**								
<30	--	--	--	--	--	--	--	--
30–50	0.13 (−0.40, 0.67)	0.59 (0.42, 0.76)^§^	0.09 (−0.22, 0.40)	0.16 (0.05, 0.27)^‡^	0.06 (−0.17, 0.30)	0.10 (−0.01, 0.21)	0.73 (0.32, 1.14)^‡^	0.31 (0.11, 0.52)^‡^
>50	0.10 (−0.42, 0.63)	0.56 (0.35, 0.76)^§^	0.44 (−0.45, 1.33)	0.26 (0.14, 0.37)^§^	0.07 (−0.22, 0.36)	0.05 (−0.12, 0.22)	0.87 (0.46, 1.28)^§^	0.31 (0.09, 0.53)^‡^
**Gender**								
Male	--	--	--	--	--	--	--	--
Female	0.06 (−0.36, 0.46)	0.20 (−0.12, 0.53)	−0.30 (−0.57, −0.04)†	−0.66 (−0.78, −0.55)^§^	0.12 (−0.41, 0.64)	−1.06 (−1.83, −0.30)^‡^	−0.71 (−1.30, −0.13)†	−0.55 (−1.02, −0.08)†
**Residence**								
Urban	--	--	--	--	--	--	--	--
Rural	0.60 (0.02, 1.17)†	−0.20 (−0.34, −0.06)^‡^	0.57 (0.16, 0.98)^‡^	0.13 (0.02, 0.23)†	0.14 (−0.01, 0.30)	−0.18 (−0.29, −0.06)^‡^	−0.05 (−0.30, 0.20)	0.19 (0.05, 0.33)^‡^
**Education**								
Less than primary	--	--	--	--	--	--	--	--
Primary	−0.04 (−0.46, 0.38)	−0.02 (−0.19, 0.16)	0.09 (−0.30, 0.49)	0.12 (0.00, 0.23)†	−0.20 (−0.49, 0.08)	−0.25 (−0.49, −0.01)†	−0.18 (−0.48, 0.13)	−0.31 (−0.48, −0.14)^§^
Secondary	0.15 (−0.38, 0.67)	0.06 (−0.14, 0.26)	-	-	−0.42 (−0.77, −0.07)†	−0.08 (−0.33, 0.17)	−0.31 (−0.79, 0.16)	−0.35 (−0.62, −0.08)^‡^
Higher than secondary	−0.19 (−1.08, 0.69)	0.05 (−0.15, 0.25)	-	-	−0.09 (−0.29, 0.11)	−0.11 (−0.21, −0.00)†	−0.20 (−0.65, 0.25)	−0.64 (−0.88, −0.40)^§^
**Occupation**								
Employed	--	--	-	-	--	--	--	--
Unemployed	0.09 (−0.69, 0.86)	−0.14 (−0.47, 0.19)	-	-	0.08 (−0.42, 0.59)	−0.12 (−0.33, 0.10)	−0.51 (−0.95, −0.06)†	−0.05 (−0.36, 0.26)
Other	0.13 (−0.19, 0.47)	0.05 (−0.08, 0.18)	-	-	0.19 (−0.08, 0.47)	−0.20 (−0.49, 0.09)	−0.04 (−0.48, 0.41)	−0.18 (−0.49, 0.12)
**Smokeless tobacco**								
Current non-user	--	--	--	--	--	--	--	--
Current user	0.14 (−0.18, 0.47)	−0.54 (−0.69, −0.40)^§^	−0.10 (−0.51, 0.32)	−0.48 (−1.10, 0.15)	0.28 (0.01, 0.55)†	−0.35 (−0.60, −0.10)^‡^	0.69 (−1.01, 2.39)	−0.32 (−1.12, 0.48)
**Waterpipe smoking**								
Current non-user	-	--	-	--	-	--	-	--
Current user	-	0.53 (0.17, 0.88)^‡^	-	0.06 (−0.13, 0.24)	-	−0.06 (−0.35, 0.23)	-	−0.52 (−0.73, −0.31)^§^
**Cigarette smoking**								
Current non-user	--	-	--	-	--	-	--	-
Current user	−0.12 (−0.46, 0.22)	-	0.31 (0.06, 0.57)†	-	−0.45 (−0.73, −0.17)^‡^	-	−0.31 (−0.56, −0.06)†	-

Note: CI: confidence interval; Volume: number of waterpipe/cigarette sessions/week;

Russia education: 1) Completed secondary or less; 2) Completed higher than secondary; Russia occupation: omitted due to very low/no respondents in other categories.

† p<0.05; ‡ p<0.01; § p<0.001.

### Further descriptive data on waterpipe users

GATS Egypt and GATS Vietnam asked further detailed questions on waterpipe. In Egypt, 97.0% (95% CI 95.7–98.4%) of respondents' previous waterpipe session was smoked using unflavoured waterpipe tobacco and 73.8% smoked it alone. 21.4% of waterpipe smokers did not share the same pipe during the session. In Vietnam, 46.1% did not share the same pipe. The mean waterpipe duration time was 42.5 minutes and 19.3 minutes for Egypt and Vietnam, respectively.

## Discussion

### Main results

Waterpipe smoking prevalence was low in all GATS countries, and in some countries (Mexico, Philippines and Thailand) virtually non-existent. Countries with waterpipe smoking prevalence above 1.0% included Russia (2.7%), Egypt (3.3%) and Vietnam (6.4%).The prevalence of dual waterpipe and cigarette smoking was 2.6% in Russia, 0.4% in Egypt, and 2.6% in Vietnam. Waterpipe smokers in Vietnam had the highest mean number of waterpipe sessions (92.9 sessions/week) with Russia the lowest (2.8 sessions/week). Compared to non-cigarette smokers, waterpipe smokers who also used cigarettes had more waterpipe smoking sessions per week in Russia (1.3 vs. 2.9, beta coefficient 0.31, 95% CI 0.06, 0.57), but less in Egypt (18.2 vs. 10.7, beta coefficient −0.45, 95% CI −0.73, −0.17) and Vietnam (102.0 vs. 79.3, beta coefficient −0.31, 95% CI −0.56, −0.06) and similar amounts in India (29.4 vs. 32.6, beta coefficient −0.12, 95% CI −0.46, 0.22).

Waterpipe smoking was significantly associated with increased age, male gender, those in rural residence and those with less than primary school education. The exception to this was Russia where younger age, urban residence and those with higher than secondary education were significant associations. In our analysis looking at the relationship between waterpipe and cigarette smoking, we found in India and Russia, cigarette smokers were more likely to be waterpipe smokers and vice versa, whereas in Egypt cigarette smokers were less likely to be waterpipe smokers with the converse being true. In Vietnam, concurrent tobacco use did not predict either cigarette or waterpipe use.

There was substantial variation in the number of weekly waterpipe sessions between countries, especially the low number of weekly sessions in Russia. The type of waterpipe used and the product smoked is likely to influence frequency of use. Adults in Egypt reported a near-exclusive use of unflavoured waterpipe tobacco, examples of which may include jurak and tumbak types [19], which are directly burnt by coal and may contain significant amounts of nicotine [9]. This waterpipe smoking method is also exhibited by India, where is it locally known as the hookah. Whilst unflavoured tobacco is also used by waterpipe smokers in Vietnam, it is an entirely different, long-stemmed instrument, which uses no charcoal and only small amounts of tobacco [20] . Waterpipe users in Russia were more educated and, in contrast to the other three countries, waterpipe was more popular in younger age groups in both men and women. These user characteristics are similar to those noted in the West among adolescents and young adults, whose pattern of use is also limited to a handful of weekly sessions [5] and more likely to involve use of flavoured tobacco [19]. These cross-country comparisons highlight the importance of understanding the cultural context of waterpipe smoking when formulating tobacco control policies.

It has been previously reported that flavoured waterpipe tobacco smoking may be a precursor to future cigarette smoking among young people [2], [3]. In India, waterpipe smoking strongly predicted both cigarette prevalence and increased levels of cigarette consumption. There was no association between waterpipe and cigarette use in Vietnam, and in fact waterpipe smokers smoked fewer cigarettes. While waterpipe smoking in Russia strongly predicted cigarette use, it did not predict increased consumption of cigarettes. Finally in Egypt, waterpipe users were less likely to be cigarette smokers, but there was no association between waterpipe smoking status and levels of cigarette consumption. These findings suggest that it may be appropriate to target waterpipe smokers as part of efforts to reduce cigarette use in some settings.

### Strengths and weaknesses

There have been few studies examining waterpipe and cigarette smoking prevalence in LMICs. A recently published study examined the prevalence of waterpipe use using the GATS data but only presented descriptive statistics and did not examine associations of dual use with cigarettes as presented here [6]. Our findings highlight important variations in waterpipe use within and between LMICs which have implications for tobacco control interventions in these settings. The study is limited by self-reported tobacco use, not validated by biochemical tests, which may underreport prevalence as a result of regional and/or country-specific norms. In some subgroup analyses, sample sizes were small which may result in high relative standard errors. The main weakness of the study is a lack of standardised collection of waterpipe prevalence data. GATS reports daily and weekly waterpipe smoking while conventional waterpipe prevalence studies enquire about past-30 day smoking as a measure for ‘current’ smokers [21]. In Western countries, prevalence studies among students have highlighted that daily and weekly flavoured waterpipe smokers may only make up 25% of all past-30 day smokers [5]. Among Arab waterpipe users in Australia, only 8.8% of past-30 day smokers were daily users [22], and in two separate studies among waterpipe café users in the USA, 33–60% were daily or weekly users [23], [24]. This distinction is important as waterpipe is frequently smoked over long sessions, typically 45 minutes, over which a user may inhale up to 30 cigarettes' worth of “tar” per session [25]. Thus a monthly, but non-weekly waterpipe user may be subject to significant tobacco intake and subsequent harm exposure but this data will not be collected on surveys such as the GATS. The other main limitation is that the GATS data are cross-sectional providing a snapshot of waterpipe smoking prevalence at point in time. Improved surveillance of waterpipe smoking in both high and low and middle income countries is required.

### Policy implications

We identified low prevalence of waterpipe smoking in most LMICs but were unable to determine whether use is increasing, as has been reported in some high income settings [4], [26]. Improving surveillance of waterpipe smoking through the GATS and other health surveys is important to monitor trends in use in key socio-demographic groups. Data on type of waterpipe used, product smoked and session frequency and length should ideally be collected alongside prevalence to gauge level of tobacco intake and inform strategies to reduce use. Tobacco control legislation appears inattentive to waterpipe smoking in countries such as the USA, where commercial waterpipe venues are exempt from smokefree legislation [27], despite the indoor air quality being arguably worse than in locations where cigarette smoking is permitted [28]. Waterpipe tobacco may also not be subject to the same level of taxation as cigarettes [29], making it an affordable method of tobacco use. Health warnings on waterpipe tobacco packaging are not compliant with Framework Convention for Tobacco Control (WHO FCTC) in many countries [30], which may contribute to reduced harm perception of waterpipe smoking among users [31].

### Conclusions

Waterpipe tobacco smoking is low in GATS countries but exhibits important differences in predictors of use that must be considered within tobacco control strategies. The relationship between waterpipe and cigarettes may vary between countries, reflecting cultural norms. Legislative efforts, including adequate health warning labelling, appropriate taxation and inclusion under the smokefree law, should be implemented on par with cigarette smoking and audited against the guidelines set out by the WHO FCTC. Meanwhile, further research is needed to understand the epidemiological course of this smoking method and associated burden of disease.

## References

[1] Akl EA, Gunukula SK, Aleem S, Obeid R, Jaoude PA, et al. (2011) The prevalence of waterpipe tobacco smoking among the general and specific populations: a systematic review. BMC public health 11.. 10.1186/1471-2458-11-244 PMC3100253 21504559

[2] Mzayek F, Khader Y, Eissenberg T, Ali RA, Ward KD, et al (2012) Patterns of water-pipe and cigarette smoking initiation in schoolchildren: Irbid longitudinal smoking study. Nicotine and Tobacco Research 14: 448–454. 22140149 10.1093/ntr/ntr234 PMC3313787

[3] Jensen PD, Cortes R, Engholm G, Kremers S, Gislum M (2010) Waterpipe use predicts progression to regular cigarette smoking among Danish youth. Substance use & misuse 45: 1245–1261. 20441461 10.3109/10826081003682909

[4] Bover Manderski MT, Hrywna M, Delnevo CD (2012) Hookah Use Among New Jersey Youth: Associations and Changes Over Time. American Journal of Health Behavior 36: 693–699. 22584096 10.5993/AJHB.36.5.11

[5] Jawad M, Abass J, Hariri A, Rajasooriar KG, Salmasi H, et al (2013) Waterpipe smoking: prevalence and attitudes among medical students in London. International Journal of Tuberculosis and Lung Disease 17: 137–140. 23232013 10.5588/ijtld.12.0175

[6] Morton J, Song Y, Fouad H, Awa FE, Abou El Naga R, et al.. (2013) Cross-country comparison of waterpipe use: nationally representative data from 13 low and middle-income countries from the Global Adult Tobacco Survey (GATS). Tobacco Control. 10.1136/tobaccocontrol-2012-050841 PMC4145417 23760609

[7] Akl E, Jawad M, Lam W, Co C, Obeid R, et al (2013) Motives, beliefs and attitudes towards waterpipe tobacco smoking: a systematic review. Harm Reduction Journal 10: 12. 23816366 10.1186/1477-7517-10-12 PMC3706388

[8] Akl EA, Gaddam S, Gunukula SK, Honeine R, Jaoude PA, et al (2010) The effects of waterpipe tobacco smoking on health outcomes: A systematic review. International Journal of Epidemiology 39: 834–857. 20207606 10.1093/ije/dyq002

[9] Neergaard J, Singh P, Job J, Montgomery S (2007) Waterpipe smoking and nicotine exposure: A review of the current evidence. Nicotine and Tobacco Research 9: 987–994. 17943617 10.1080/14622200701591591 PMC3276363

[10] Benowitz NL, Henningfield JE (1994) Establishing a Nicotine Threshold for Addiction -- The Implications for Tobacco Regulation. New England Journal of Medicine 331: 123–125. 7818638 10.1056/NEJM199407143310212

[11] Martin R, Safaee S, Somsamouth K, Mounivong B, Sinclair R, et al (2013) Mixed Methods Pilot Study of Sharing Behaviors among Waterpipe Smokers of Rural Lao PDR: Implications for Infectious Disease Transmission. International Journal of Environmental Research and Public Health 10: 2120–2132. 23708049 10.3390/ijerph10062120 PMC3717727

[12] Blank MD, Brown KW, Goodman RJ, Eissenberg T (2013) An Observational Study of Group Waterpipe Use in a Natural Environment. Nicotine & Tobacco Research. 10.1093/ntr/ntt120 PMC3864492 23943842

[13] WHO (2005) World Health Organisation TobReg. Water-pipe tobacco smoking: health effects, research needs and recommended actions by regulators. Geneva, Switzerland.

[14] Jawad M, Wilson A, Lee JT, Jawad S, Hamilton F, et al.. (2013) Prevalence and predictors of waterpipe and cigarette smoking among secondary school students in London. Nicotine & Tobacco Research (in press) DOI: 10.1093/ntr/ntt103. 10.1093/ntr/ntt103 23884320

[15] Rath JM, Villanti AC, Abrams DB, Vallone DM (2012) Patterns of Tobacco Use and Dual Use in US Young Adults: The Missing Link between Youth Prevention and Adult Cessation. Journal of environmental and public health. 10.1155/2012/679134 PMC3361253 22666279

[16] Almerie MQ, Matar HE, Salam M, Morad A, Abdulaal M, et al (2008) Cigarettes and waterpipe smoking among medical students in Syria: A cross-sectional study. International Journal of Tuberculosis and Lung Disease 12: 1085–1091. 18713509 PMC2553239

[17] Nazar GP, Lee JT, Glantz SA, Arora M, Pearce N, et al (2014) Association between being employed in a smoke-free workplace and living in a smoke-free home: Evidence from 15 low and middle income countries. Preventive Medicine 59: 47–53. 24287123 10.1016/j.ypmed.2013.11.017 PMC3898883

[18] Kalsbeek WD, Bowling JM, Hsia J, Mirza S, Palipudi KM, et al.. (2010) The Global Adult Tobacco Survey (GATS): Sample Design and Related Methods.

[19] Knishkowy B, Amitai Y (2005) Water-pipe (narghile) smoking: an emerging health risk behavior. Pediatrics 116: e113–e119. 15995011 10.1542/peds.2004-2173

[20] She J, Yang P, Bai C (2012) Chinese Waterpipe Smoking: A New Risk Factor for Lung Cancer and COPD? Chest 142..

[21] Maziak W, Ward KD, Afifi RA, Eissenberg T (2005) Standardizing questionnaire items for the assessment of waterpipe tobacco use in epidemiological studies. Public Health 119: 400–404. 15780328 10.1016/j.puhe.2004.08.002

[22] Carroll T, Poder N, Perusco A (2008) Waterpipe tobacco smoking: An important public health issue. Australian and New Zealand Journal of Public Health 32: 490–491. 18412692 10.1111/j.1753-6405.2008.00198.x

[23] Smith-Simone S, Maziak W, Ward K, Eissenberg T (2008) Waterpipe tobacco smoking: Knowledge, attitudes, beliefs, and behavior in two U.S. samples. Nicotine and Tobacco Research 10: 393–398. 18236304 10.1080/14622200701825023 PMC3215239

[24] Ward KD, Eissenberg T, Gray JN, Srinivas V, Wilson N, et al (2007) Characteristics of U.S. waterpipe users: A preliminary report. Nicotine and Tobacco Research 9: 1339–1346. 18058352 10.1080/14622200701705019

[25] Shihadeh A (2003) Investigation of mainstream smoke aerosol of the argileh water pipe. Food and Chemical Toxicology 41: 143–152. 12453738 10.1016/s0278-6915(02)00220-x

[26] Smith JR, Edland SD, Novotny TE, Hofstetter CR, White MM, et al (2011) Increasing hookah use in California. American Journal of Public Health 101: 1876–1879. 21852640 10.2105/AJPH.2011.300196 PMC3222344

[27] Primack BA, Hopkins M, Hallet C, Carroll MV, Zeller M, et al (2012) US Health Policy Related to Hookah Tobacco Smoking. American Journal of Public Health 102: e47–e51. 22827447 10.2105/AJPH.2012.300838 PMC3482044

[28] Cobb CO, Vansickel AR, Blank MD, Jentink K, Travers MJ, et al (2013) Indoor air quality in Virginia waterpipe cafes. Tobacco Control 22: 338–343. 22447194 10.1136/tobaccocontrol-2011-050350 PMC3889072

[29] Morris DS, Fiala SC, Pawlak RP (2012) Opportunities for Policy Interventions to Reduce Youth Hookah Smoking in the United States. Preventing chronic disease 9.. 10.5888/pcd9.120082 PMC3505114 23153772

[30] Nakkash R, Khalil J (2010) Health warning labelling practices on narghile (shisha, hookah) waterpipe tobacco products and related accessories. Tobacco Control 19: 235–239. 20501497 10.1136/tc.2009.031773 PMC2989164

[31] Roskin J, Aveyard P (2009) Canadian and English students' beliefs about waterpipe smoking: a qualitative study. BMC public health 9.. 10.1186/1471-2458-9-10 PMC2628878 19134220

